# An F2 Pig Resource Population as a Model for Genetic Studies of Obesity and Obesity-Related Diseases in Humans: Design and Genetic Parameters

**DOI:** 10.3389/fgene.2013.00029

**Published:** 2013-03-18

**Authors:** Lisette J. A. Kogelman, Haja N. Kadarmideen, Thomas Mark, Peter Karlskov-Mortensen, Camilla S. Bruun, Susanna Cirera, Mette J. Jacobsen, Claus B. Jørgensen, Merete Fredholm

**Affiliations:** ^1^Department of Veterinary Clinical and Animal Sciences, Faculty of Health and Medical Sciences, University of CopenhagenCopenhagen, Denmark

**Keywords:** animal model, F2 design, obesity, diabetes, heritabilities, genetic correlations, genetic predictions

## Abstract

Obesity is a rising worldwide public health problem. Difficulties to precisely measure various obesity traits and the genetic heterogeneity in human have been major impediments to completely disentangle genetic factors causing obesity. The pig is a relevant model for studying human obesity and obesity-related (OOR) traits. Using founder breeds divergent with respect to obesity traits we have created an F2 pig resource population (454 pigs), which has been intensively phenotyped for 36 OOR traits. The main rationale for our study is to characterize the genetic architecture of OOR traits in the F2 pig design, by estimating heritabilities, genetic, and phenotypic correlations using mixed- and multi-trait BLUP animal models. Our analyses revealed high coefficients of variation (15–42%) and moderate to high heritabilities (0.22–0.81) in fatness traits, showing large phenotypic and genetic variation in the F2 population, respectively. This fulfills the purpose of creating a resource population divergent for OOR traits. Strong genetic correlations were found between weight and lean mass at dual-energy x-ray absorptiometry scanning (0.56–0.97). Weight and conformation also showed strong genetic correlations with slaughter traits (e.g., *r*_g_ between abdominal circumference and leaf fat at slaughtering: 0.66). Genetic correlations between fat-related traits and the glucose level vary between 0.35 and 0.74 and show a strong correlation between adipose tissue and impaired glucose metabolism. Our power calculations showed a minimum of 80% power for QTL detection for all phenotypes. We revealed genetic correlations at population level, for the first time, for several difficult to measure and novel OOR traits and diseases. The results underpin the potential of the established F2 pig resource population for further genomic, systems genetics, and functional investigations to unravel the genetic background of OOR traits.

## Introduction

Obesity is a growing worldwide public health problem with a prevalence that has almost doubled between 1980 and 2008 (World Health Organization, 2012), resulting in one of the most significant public health challenges of the twenty-first century. In 2008, more than 1.4 billion adults were overweight and more than 0.5 billion were obese. Obesity has been defined by O’Rahilly (2009) as “a state in which the total amount of triglyceride stored in adipose tissue is abnormally increased.” As a result, several other organs and important metabolic systems are dysregulated in obese individuals (Goossens, 2008). Obesity is associated with several severe diseases such as type 2 diabetes and cardiovascular diseases, which are all closely related to each other and resulting from the interaction between genetic and environmental factors (Bener et al., 2005). The recent increase in the worldwide prevalence of obesity has increased the need for knowledge about the genetic and biological background of obesity and related metabolic diseases. A deeper insight into the genetic background and, in turn, novel metabolic molecular pathways will help to explain why some people are susceptible to obesity whereas others remain lean. Consequently, this could lead to improvement of prevention, therapy, and treatment of obesity and obesity-related (OOR) diseases (Farooqi and O’Rahilly, 2007).

Many years of research has revealed the difficulty in pinpointing the genetic background of OOR traits and diseases. Several genome-wide association studies (GWAS) have been performed to identify common genetic variants contributing to OOR traits and diseases. Using a GWAS-approach, 6–11% of the genetic variance could be explained in body mass index (BMI) by the identification of 32 loci, while heritability was estimated at 40–70% (Speliotes et al., 2010) and 25–30% of the genetic variance could be explained in blood lipids by the identification of 95 loci (Teslovich et al., 2010). For type 2 diabetes at least 10 loci were identified using GWAS strategies (Scott et al., 2007; Sladek et al., 2007), but their contribution to individual risk prediction of type 2 diabetes is relatively small. In human studies of these traits, it has been shown to be extremely difficult to pinpoint the most important biological pathways involved in complex traits due to confounding between genetics and environment and due to genetic heterogeneity. Moreover, precise measurements on various OOR traits in human research are often difficult, very expensive, and in some cases even impossible, resulting in the inability to detect the total genetic variance. A complex trait such as obesity is a combination of numerous phenotypic conditions. By dissecting them into endophenotypes, the phenotype can be more accurately defined which will improve the genetic detection of diseases (Bougnères, 2002). An animal model will not only help reduce costs substantially but also helps control the environment. It has the potential to create a large dataset of various unique phenotypic measurements for genetic research to increase knowledge about the genetic and biological background of OOR traits.

Expression studies of several genes involved in glucose metabolism in adipose tissues have produced markedly different results in humans and rodents hindering the translation from findings in rodent models to human biology (Arner, 2005). The pig resembles humans more than rodents, as the pig shares metabolic and digestive features as well as cardiovascular systems and other anatomical and physiological features with humans (Spurlock and Gabler, 2008). The recent annotation of the porcine genome (Groenen et al., 2012) as well as QTL and selective sweep analysis (Rubin et al., 2012) has demonstrated that homologous variants are present in human and pigs. Moreover, it has been shown that human obesity genes, such as FTO and MC4R, have homologous variants in pigs (Kim et al., 2000; Fontanesi et al., 2009). As fatness in pigs is an economically important trait in pig production, several studies have focused on the genetics of fatness in commercial pig lines. A large study on fatness traits in pigs uncovered 663 genes with expression profiles that were significantly correlated with the trait “fat area” (in cm^2^, measured between the 13th and 14th rib) as well as biologically relevant pathways and networks (Ponsuksili et al., 2011). Several QTL and GWAS studies have been conducted using pigs from the same breeds used as founders in the studied population. In the Yorkshire pigs, e.g., several micro-satellite markers were significantly associated with average daily gain and backfat thickness (Kim et al., 2006). In a Duroc × Pietrain F2 population, several QTLs were significantly associated with serum lipids, including a large QTL on SSC1 (Uddin et al., 2011). This has also been reported to be in association with average daily gain and carcass composition in the same population (Liu et al., 2007). Recently, a region on SSC6 for backfat thickness was detected in Duroc pigs (Okumura et al., 2012) and in commercial pigs several QTL regions were detected in association with body composition traits in commercial pigs (Large White and Large White × Landrace crosses) (Fan et al., 2011). As per the review of Bellinger et al. (2006) pigs are a useful model for type 2 diabetes, as they show type 2 diabetes related characteristics. From this review, it can be concluded that both the Göttingen minipig and the Yorkshire pigs are valuable sources to investigate the genetic and biological background of type 2 diabetes.

Another great advantage of using the pig as a model in genetic research is the possibility to create a population segregating for the traits of interest. That is, by mating genetically and phenotypically divergent individuals and subsequently intercrossing the offspring, an informative resource population can be established for genetic studies. An important prerequisite for a successful outcome of exploiting a resource population is the ability to precisely define and measure phenotypes, endophenotypes, and intermediate phenotypes (Kadarmideen et al., 2006) (e.g., transcriptome or proteome) and control the environment and diet in an experimental setting. Such resource populations allow not only genetic (GWAS) studies but also systems genetics and biology studies where genetic variations in intermediate phenotypes can be studied by integrative approaches (Kadarmideen et al., 2011; Kogelman et al., 2011).

An F2 pig resource population was established to increase knowledge about the genetic and biological background of OOR traits because of the above-mentioned arguments. It is well accepted that before conducting quantitative trait loci (QTL) mapping or GWAS in resource populations, a thorough knowledge on (additive) genetic variance of each trait and (genetic) relationships amongst them are investigated, particularly if such parameters for novel traits have not yet been reported elsewhere. For instance, genetic parameters for 16 different trypanotolerance traits were estimated (Van Der Waaij et al., 2003) for detection of QTLs for these trypanotolerance traits in a F2 design (Hanotte et al., 2003). In this paper, we report the establishment of the F2 pig resource population and quantify its genetic make-up by estimating heritabilities of 36 different (and partly novel) OOR traits and genetic and phenotypic correlations amongst them.

## Materials and Methods

### Experimental design

An F2 population was created using Danish production pig breeds, i.e., purebred Yorkshire (YY) and Duroc (DD) sows from a DanBred breeding herd and Göttingen minipig (MM) boars from Ellegaard A/S in the parental generation. The production pig breeds have been selected for leanness and growth among other traits during the last 60 years, whereas Göttingen minipigs are prone to obesity and bred principally for their small size and ease of handling. They are also known to share the metabolic impairments seen in obese humans (Johansen et al., 2001). For the parental animals two different maternal lines were used to ensure a higher likelihood of detecting narrow QTL regions, as it is expected that many of the same genes are fixed in the different breeds, but the LD structure will differ between them.

**Figure 1:**
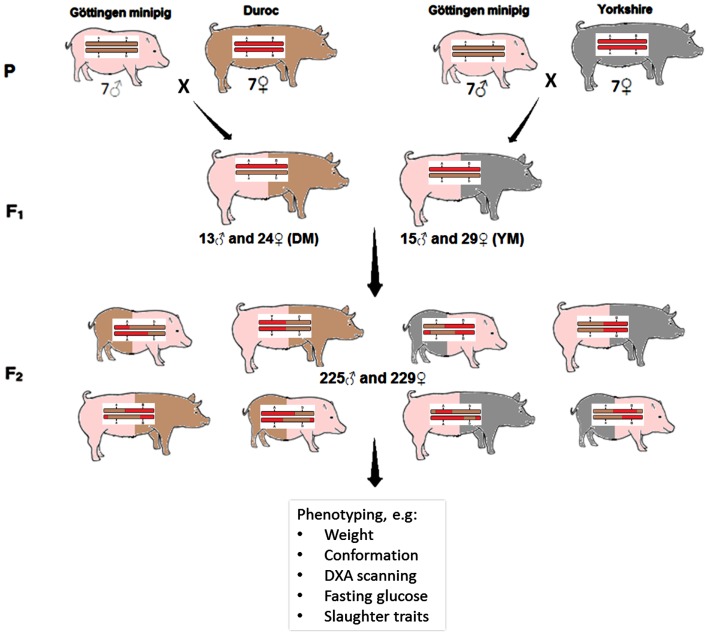
**Overview of the experimental design**. The resource population consisting of 454 F2 pigs was created and extensively phenotyped using a cross between lean production pigs (Yorkshire/Duroc) × Göttingen minipig.

Seven female Duroc pigs and seven female Yorkshire pigs were artificially inseminated with 14 male Göttingen minipigs (28 F0 animals), resulting in 14 full-sib F1 families. In total, 127 F1 animals were produced: 60 males and 67 females, from which 81 F1 animals were randomly selected to produce the F2 generation. A total of 29 Minipig-Yorkshire (MY) and 24 Minipig-Duroc (MD) gilts were mated to respectively 15 MY and 13 MD non-litter mate boars (each boar used once or twice) to produce the F2 generation, so all F2 animals were a combination of Minipig × Yorkshire or Minipig × Duroc (Figure 1).

The F2 animals were produced at the research farm, University of Copenhagen Tåstrup, Denmark. Animal care and maintenance have been conducted according to the Danish “Animal Maintenance Act” (Act 432 dated 09/06/2004). A total of 474 piglets were born in three different groups, specified by season of birth (SOB), whereby the first SOB consisted of approximately 250 piglets born in June or July, the second SOB consisted of approximately 125 piglets born in August, September, or October, and the third SOB consisted of approximately 100 piglets born in December or January. Litters in the F2 generation consisted on average of eight piglets per litter and ranged from 2 to 12 born piglets. Piglets were weaned at 6–8 weeks of age. In total, the F2 generation consisted of 474 born piglets, 469 of which were still alive at 5 weeks of age and used in further analyses. The complete pedigree consists of 563 animals.

### Collection of phenotypes

**Figure 2:**
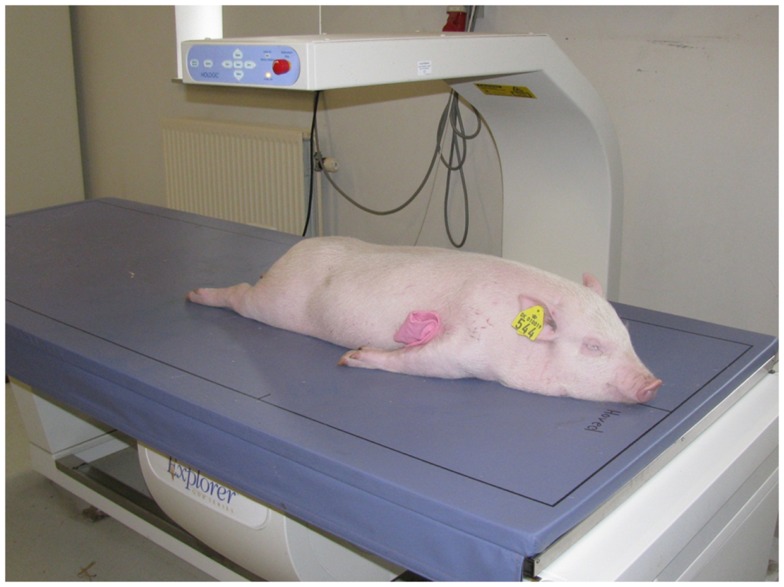
**A dual-energy x-ray absorptiometry (DXA) scan of one of the pigs at approximately 2.5 months old**.

**Figure 3:**
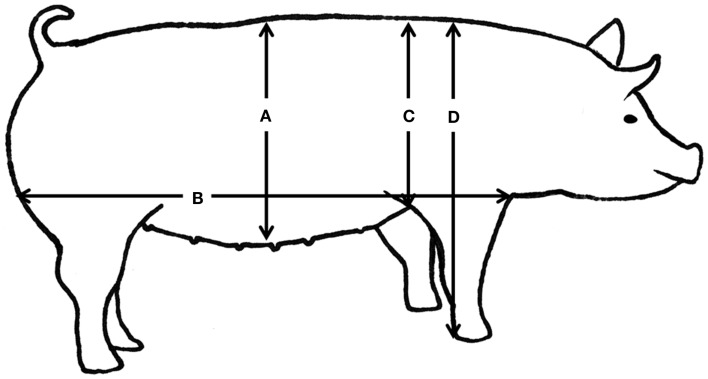
**Body conformation measurements in the pig**. Body conformation measurements in the pig including abdominal circumference at the navel **(A)**, the length of the body from rump to the front of the shoulder blade **(B)**, shoulder height **(C)**, and thorax height **(D)**.

Intensive phenotypic recording was performed on all pigs from birth to slaughter. Body weight was measured at birth and at the following approximate time points: 2 weeks (13–16 days), 5 weeks (34–38 days), 2 months (64 ± 11 days), 125 days (115 ± 27 days), and 7 months (220 ± 45 days). After weaning, at approximately 2 months of age (64 ± 11 days), the piglets were anesthetized with 1 ml/10 kg of a mixture containing zolazepam and tiletamin (Zoletil^®^ 50 Vet., ChemVet, Denmark), ketamin (Ketaminol^®^ Vet. 100 mg/ml, Intervet, Denmark), detomidin (Domitor 1 mg/ml, Pfizer Animal Health, Denmark), and buthorphanol (Torbugesic^®^, 10 mg/ml, Scanvet, Denmark). This full sedation is approved and granted by the Experimental Animal Inspectorate on July 1 2011 (approval number2007/561-1434). Body composition was determined using dual-energy x-ray absorptiometry (DXA) scanning (Hologic Explorer, Santax Medico, Aarhus, Denmark). The scanning was performed with the pigs in ventral recumbency with the front and hind limbs stretched backwards (Figure 2). Using the differential attenuation of low (38 keV) and high-energy (70-keV) x-rays the body composition of fat and other soft tissues was estimated. The analysis of body composition was performed using the scanner software package, providing measurements of fat mass, lean mass, total mass, fat percentage, bone mineral content (BMC), and bone mineral density (BMD). Previous research indicated that those DXA measurements are very accurate in comparison with chemical analysis (Mitchell et al., 1998). Subsequently, blood was collected from the jugular vein and the following body conformation traits were measured (Figure 3): abdominal circumference (Figure 3A), length of the body from rump to the front of the shoulder blade (Figure 3B), shoulder height (Figure 3C), and thorax height (Figure 3D).

At approximately 2.5 months of age, pigs were transported to a production pig farm (Ringsted, Denmark) and placed in pens (20 m^2^) with an average of 15 piglets of the same sex per pen. Pigs were fed *ad libitum* with standard production pig feed (Classic, dlg.dk) and with free access to water. All pigs were weighed two to four times within the period from 2.5 months to slaughter at approximately 80–100 kg. At the time of weighing, body conformation measurements were recorded (Figure 3). Fasting glucose was measured on a subset of the pigs (146 animals) fasted for 24 h at the age of 6–8 months. The measurements were performed with an OneTouch UltraSmart Blood Glucose Monitoring System (Lifescan, Inc., Ireland) on blood collected from the jugular vein. The pigs were slaughtered at a commercial slaughterhouse. The pigs were euthanized by bleeding after electrical stunning and the following phenotypes were recorded: weight of leaf fat, weight of the great omentum (272 animals), and weight of mesenteric fat as measured from an 8 cm diameter large section of the omentum in the triangle between ileum and cecum (234 animals). A Fat-o-Meat’er (Carometec A/S, Herlev, Denmark) was used to measure thickness of back fat and meat in millimeters. Measurements were taken between the third and fourth lumbar vertebra (position 1) and between the third and fourth last rib (position 2), both at 8 cm from midline. Meat percentage was calculated as:

$$\hat{Y}=66.7393-0.2655*X1-0.5432*X2+0.0838*X3$$

where Ŷ is the meat in percentage, *X*1 is the backfat measurement at position 1, *X*2 is the backfat measurement at position 2 and *X*3 is the muscle thickness at position 2. The Fat-o-Meat’er detects changes in light reflectance to determine shifts between fat and muscle. However, it is known that fat percentage in pigs is positively correlated to the reflectance (Hodgson et al., 1991) resulting in inaccurate measurements in very obese animals. Therefore, Fat-o-Meat’er measurements were set as missing values when reflectance was above 65 (equal to 2.5σ above the mean), thereby excluding inaccurate measurements. Furthermore, carcass weight (excl. skin head and leaf fat) was also recorded.

For subsequent data analysis some variables were calculated using the recorded measurements. Average daily gain was estimated over the whole growth period (from birth to approximately 7 months of age), and as daily gain pre- and post-weaning. In human studies, BMI is a commonly used indicator of obesity (Saunders et al., 2007). It was calculated as weight/(height)^2^ and therefore in pigs it has been calculated as weight/(length)^2^, whereby length is the length of the body from rump to the front of the shoulder blade (Figure 3B). Bergman et al. (2011) defined a new parameter to estimate percentage of adiposity more accurately, the body adiposity index (BAI). BAI in humans is proportional to the hip circumference/(height)^1.5^. Therefore, in this project it has been calculated as abdominal circumference/(length)^1.5^, where the abdominal circumference is illustrated in Figure 3A.

In total 15F2 animals were removed from the dataset, due to illness or death, which created a F2 resource population of 454 animals.

### Statistical analysis and power calculations

Data exploration of all variables was performed to detect outliers, distributions, and relations among variables. Exploration of distribution showed that DXA traits (DXA fat, DXA lean, DXA total, DXA%fat, BMC, and BMD) were not normally distributed and therefore, the first four were transformed using log_10_ transformation and the last two were transformed using a square-root transformation, respectively, to approach the normal distribution. Quantile–quantile plots indicated that the residuals of transformed variables were approximately normally distributed. All fixed effects were initially analyzed using the GLM procedure of SAS (SAS Version 9.3, 2012) to determine significant fixed effects with respect to all analyzed variables. According to these preliminary analyses, sex, SOB, and age (at time of measurement) were taken into account as fixed effects. For the variables DXA fat, DXA lean, DXA total, SLfat, SLfat_om, and SLfat_int, the length of the animal at the relevant age was also taken into account as fixed effect. The length of the animal is taken into account, because of the difference in conformation of the production pig (long and lean) and the Göttingen minipig (short and thick). Table A1 in Appendix presents the solutions for all fixed effects for the different variables. In addition, in order to detect whether there was sufficient power to detect QTL for the phenotypes under study, we have conducted statistical power/sample size calculations at very conservative assumptions, using qtlDesign R software (Sen et al., 2007). We assumed QTL explains a small (0.5%) to large (15%) proportion of the phenotypic variance at moderate and slightly high heritabilities (of 0.25 and 0.50) with an average distance between SNP markers less than 1 cm. The empirical methods of calculating power for QTL mapping for categorical traits (Kadarmideen et al., 2000) was also adapted for our design to show power on underlying liability scale.

### Variance components estimation

An univariate animal model was fitted to the data using ASReml (Gilmour et al., 2009) to estimate variance components and heritabilities for each trait considered. The model used for variance component estimations was:

$$\begin{array}{l} Y_{\text{ijkl}}=\mu +{\text{GENDER}}_{i}+{\text{SOB}}_{j}+b_{k}(\text{AGE})+b_{l}(\text{LENGTH})\\ \;+{\text{ANIMAL}}_{m}+e_{\text{ijklm}} \end{array}$$

where *GENDER* is the fixed effect of the sex *i* of the animal (two classes), *SOB* is the fixed effect of the SOB *j* when the pigs were born (three classes), *AGE* is the age *k* of the pig in days at the time of measurement as covariate, and *b_k_* is the associated fixed regression coefficient. *LENGTH* is the length of the animal in cm at the time of measuring as a covariate and *b_l_* is the associated fixed regression coefficient. The latter effect was only included for fat mass, lean mass, total mass (at DXA scanning) and weight of leaf fat at slaughtering. Random effects included were *ANIMAL*, the additive genetic effect of the animal *l*, and *e*, the error term. The following (co)variances among random effects were fitted:

$$\mathrm{var}\;\left(\begin{array}{c} \text{a}\\ \text{e} \end{array}\right)=\left(\begin{array}{cc} \text{A}\sigma_{\text{a}}^{2} & 0\\ 0 & I\sigma_{e}^{2} \end{array}\right)$$

where **A** is the additive genetic relationship matrix and **I** is an identity matrix. The animal and residual terms were assumed to be $a\sim N\left(0,\;\text{A}\sigma_{\text{a}}^{2}\right);\;e\sim N\left(0,\;\text{I}\sigma_{\text{e}}^{2}\right)$ where $\sigma_{a}^{2}$ is the additive genetic variance and $\sigma_{e}^{2}$ the error variance.

### Bivariate models for estimation of genetic and phenotypic correlation

In order to estimate heritabilities and genetic and phenotypic correlations, we fitted a series of bivariate animal models for all combinations of traits. The bivariate animal model can be represented as follows:

$$\left(\begin{array}{c} y_{1}\\ y_{2} \end{array}\right)=(\begin{array}{cc} X_{1} & 0\\ 0 & X_{2} \end{array})\;(\begin{array}{c} b_{1}\\ b_{2} \end{array})\;+(\begin{array}{cc} Z_{1} & 0\\ 0 & Z_{2} \end{array})\;(\begin{array}{c} a_{1}\\ a_{2} \end{array})\;+(\begin{array}{c} e_{1}\\ e_{2} \end{array})\;$$

where **y_1_** and **y_2_** are vectors of observations for the concerning traits, **b_1_** and **b_2_** are vectors of fixed effects for the concerning traits, **a_1_** and **a_2_** are vectors of random animal effects for the concerning traits, **X_1_** and **X_2_** the incidence matrices relating records of the concerning traits to fixed effects, and **Z_1_** and **Z_2_** are incidence matrices relating observations with random animal effects. The expectations and variance are:

$$E\left(\begin{array}{c} y_{1}\\ y_{2} \end{array}\right)=(\begin{array}{cc} X_{1} & 0\\ 0 & X_{2} \end{array})\;\left(\begin{array}{c} b_{1}\\ b_{2} \end{array}\right)$$

and

$$\text{Var }\left(\begin{array}{c} a_{1}\\ a_{2}\\ e_{1}\\ e_{2} \end{array}\right)=\left(\begin{array}{cccc} A\sigma_{a1}^{2} & A\sigma_{a12}^{2} & 0 & 0\\ A\sigma_{a21}^{2} & A\sigma_{a2}^{2} & 0 & 0\\ 0 & 0 & I\sigma_{e1}^{2} & I\sigma_{e12}^{2}\\ 0 & 0 & I\sigma_{e21}^{2} & I\sigma_{e2}^{2} \end{array}\right)$$

where $\sigma_{a1}^{2}$ and $\sigma_{a2}^{2}$ are additive genetic variances for traits 1 and 2, $\sigma_{a12}^{2}=\sigma_{a21}^{2}$ is the additive genetic covariance between traits 1 and 2, $\sigma_{e1}^{2}=\sigma_{e2}^{2}$ are residual variances for traits 1 and 2, and $\sigma_{e12}^{2}=\sigma_{e21}^{2}$ is the residual covariance between traits 1 and 2. **A** and **I** are as described above, while **a** and **e** were assumed to be normally distributed with a mean of zero and (co)variances as specified above.

All analyses were implemented using ASReml (Gilmour et al., 2009). Heritabilities, genetic correlations, and phenotypic correlations were calculated from the estimated (co-) variance components. Estimation of heritability with the lowest standard error (univariate or bivariate) was used as heritability for the concerning trait.

## Results and Discussion

### Descriptive statistics of the F2 pig resource population

We have created an F2 pig resource population with an extensive phenotypic variation for OOR traits using Göttingen minipigs (genetically predisposed to obesity) and Duroc or Yorkshire pigs (genetically selected for leanness) as founder breeds. This resource population has been comprehensively phenotyped for several OOR traits with the aim to conduct, among other things, QTL mapping, and GWAS. Table 1 shows descriptive statistics of all analyzed traits directly related to OOR, while Table 2 shows descriptive statistics of all analyzed OOR traits indirectly related to OOR in the F2 resource population. Both tables show the mean, SD, range, and coefficient of variation (CV). It is clear from the descriptive statistics that there is substantial phenotypic variation of the traits measured within the pedigree, as illustrated by the high CV values (from 7.5 to 42.4%). In particular, CV values for the fatness traits are very high (f.e., 42.2% for SLfat), confirming that the design of the pedigree has fulfilled the purpose of creating a resource population with extensive phenotypic variation. Bahelka et al. (2007) reported CV’s for different slaughter traits which were all lower than those reported in our F2 pig resource population, e.g., 8.53% for carcass weight, 19.08% for backfat, and 23.05% for fat (kg). It is also important to note that fasting glucose levels (FGLs) ranged from 1.8 to 11.15 mmol/L with a CV of 22.5%, indicating that metabolic parameters involved in, for instance, type 2 diabetes show a wide phenotypic variation in the resource population. Hence, we believe the established F2 resource population is an excellent model suitable for further genetic, genomic, systems genetics, and functional investigations of OOR traits.

**Table 1:** **Summary of all traits directly associated with obesity and obesity-related traits (mean, range, and SD) with their abbreviations**.

Trait		Unit	Abbreviation	*N*	Mean	SD	Minimum	Maximum	CV (%)
**SLAUGHTER TRAITS**									
	Carcass weight	kg	SLcw	358	56.33	11.77	30.00	117.00	20.89
	Meat	mm	Slmeat	330	52.15	7.74	24.00	85.00	14.85
	Meat percentage	%	SL%meat	330	43.39	6.65	28.00	59.10	15.33
	Weight leaf fat	kg	SLfat	396	2.59	1.10	0.32	8.64	42.40
	Backfat position 1^1^	mm	SLbf1	330	31.90	8.54	10.00	54.00	26.77
	Backfat position 2^2^	mm	SLbf2	330	35.44	9.12	15.00	64.00	25.74
	Omental fat	kg	SLfat_om	257	352.72	145.28	68.68	893.83	41.19
	Intestinal fat	kg	SLfat_int	219	19.88	8.32	4.65	56.92	41.85
**DXA TRAITS**									
	Fat mass	kg	DXA fat	438	22.92	8.53	6.91	57.65	37.24
	Lean mass	kg	DXA lean	438	100.42	37.33	34.32	272.97	37.18
	Total mass	kg	DXA total	438	123.34	44.93	43.26	330.62	36.43
	Fat (%)	%	DXA%fat	438	18.64	2.87	11.58	28.48	15.41
Glucose	Fasting level	mmol/L	FGL	146	4.50	2.28	1.80	11.15	50.67

*^1^Backfat measured between third and fourth lumbar vertebra, 8 cm off midline*.

*^2^Backfat measured between third and fourth last rib, 8 cm off midline*.

**Table 2:** **Summary of all traits indirectly associated with obesity and obesity-related traits (mean, range, and SD) with their abbreviations**.

Trait		Unit	Abbreviation	*N*	Mean	SD	Minimum	Maximum	CV (%)
**WEIGHT TRAITS**									
	At birth	kg	WTbirth	436	0.82	0.17	0.39	1.70	20.10
	At 2 weeks	kg	WT2w	439	2.73	0.52	0.81	5.60	19.09
	At 5 weeks	kg	WT5w	438	5.94	1.37	1.98	10.54	23.15
	At ±2 months^4^	kg	WT2m	439	12.45	4.52	4.40	33.70	36.27
	At ±125 days^5^	kg	WT125d	287	36.80	11.05	8.50	63.00	30.01
	At ±7 months^6^	kg	WT7m	405	94.45	17.51	45.50	189.00	18.54
**DAILY GAIN**									
	Average	kg/day	ADG	403	0.44	0.08	0.23	0.71	18.94
	Pre-weaning	kg/day	DGI	432	0.18	0.05	0.06	0.32	28.31
	Post-weaning	kg/day	DGII	401	0.55	0.13	0.24	1.02	23.61
**CONFORMATION TRAITS**									
Length	At ±2 months	cm	LEN2m	439	48.52	5.89	34.50	69.00	12.14
	At ±7 months	cm	LEN7m	404	84.40	6.37	65.00	108.00	7.54
Abd. Circ.^1^	At ±2 months	cm	ABD2m	439	59.97	7.75	40.50	88.00	12.92
	At ±7 months	cm	ABD7m	404	122.70	10.56	87.00	160.00	8.60
Shoulder height	At ±2 months At ±7 months	cm cm	SH2m SH7m	439 268	39.89 65.34	4.37 5.04	24.00 45.00	55.00 79.00	10.97 7.71
	At ±7 months	cm	SH7m	268	65.34	5.04	45.00	79.00	7.71
Thorax height	At ±2 months	cm	TH2m	439	22.86	3.70	14.00	62.00	16.19
	At ±7 months	cm	TH7m	403	52.39	5.69	34.00	69.00	10.85
BMI^2^	At ±2 months	kg/cm^2^	BMI2m	439	51.08	7.42	31.86	84.32	14.53
	At ±7 months	kg/cm^2^	BMI7m	403	132.86	20.71	74.79	217.98	15.59
BAI^3^	At ±2 months	cm/cm^1.5^	BAI2m	439	0.18	0.02	0.13	0.24	9.04
	At ±7 months	cm/cm^1.5^	BAI7m	404	0.16	0.02	0.11	0.22	11.60
Bone mineral	Content	g/cm^3^	BMC	438	301.42	94.43	112.86	697.70	31.33
	Density	g/cm^3^	BMD	438	0.53	0.08	0.34	0.79	14.52

*^1^Abd. Circ, abdominal circumference*.

*^2^BMI, body mass index, calculated as [weight/(length)^2^]*.

*^3^BAI, body adiposity index, calculated as [(abdominal circumference)/(length)^1.5^]*.

*^4^Age at ±2 months is within a range of 46–103 days*.

*^5^Age at ±125 days is within a range of 86–215 days*.

*^6^Age at ±7 months is within a range of 150–458 days*.

Dual-energy x-ray absorptiometry measurements of fat percentage in the F2 resource population showed an average of 18.6% (SE = 2.9). Previous research showed that DXA is an accurate method for estimating body fat, lean mass, and BMC in pork carcasses (Suster et al., 2004). Mitchell et al. (1998) reported fat percentage of 11.43(±3.35) kg, which is slightly lower than the estimate of fat percentage in our population (18.64 ± 2.87 kg). The BMC was higher (338 ± 17) than in our research population (301 ± 94).

### Heritabilities

Heritabilities are presented in Table 3 and all estimates were statistically highly significant. In general, most estimated heritabilities were high, and higher than previous reports for some traits studied in field populations.

**Table 3:** **Heritabilities and standard errors estimated with the bivariate model using all 563 animals (454 with records)**.

Trait^1^	*h*^2^	s.e.^2^	Trait^1^	*h*^2^	s.e.^2^
SLcw	0.54	0.14	WT2m	0.78	0.14
SLmeat	0.14	0.10	WT125d	0.67	0.17
SL%meat	0.18	0.10	WT7m	0.39	0.12
SLfat	0.23	0.12			
SLbf1	0.22	0.11	LEN2m	0.52	0.14
SLbf2	0.23	0.13	LEN7m	0.26	0.11
SLfat_om	0.52	0.15	ABD2m	0.49	0.13
SLfat_int	0.08	0.07	ABD7m	0.28	0.11
			SH2m	0.72	0.14
DXA fat	0.43	0.13	SH7m	0.30	0.13
DXA lean	0.71	0.14	TH2m	0.82	0.14
DXA total	0.67	0.15	TH7m	0.38	0.12
DXA%fat	0.57	0.14			
			BMI2m	0.56	0.14
FGL	0.49	0.22	BMI7m	0.23	0.11
			BAI2m	0.58	0.14
ADG	0.54	0.14	BAI7m	0.31	0.13
DGI	0.81	0.14			
DGII	0.60	0.14	BMC	0.76	0.15
			BMD	0.92	0.16

*^1^For abbreviations and units of measurements see Table 1*.

*^2^Standard error*.

There was not sufficient information in the data to analyze weight at birth, at 2 weeks of age and at 5 weeks of age. Heritability was high for weight at 2 months (0.78, SE = 0.14) and at 125 days (0.67, SE = 0.17), and slightly lower at 7 months (0.39, SE = 0.12). Huisman et al. (2002) used two different random regression models to estimate heritability of field data of production pigs using three time points to measure: approximately 73, 135, and 190 days. This resulted in heritabilities of 0.14–0.53, 0.17–0.61, and 0.23–0.56, respectively. Johnson and Nugent (2003) estimated heritabilities for weight slightly lower, i.e., between 0.12 and 0.27 at 100 days of age for different commercial production breeds (Landrace, Yorkshire, Duroc, and Hampshire), and between 0.15 and 0.27 at 177 days of age. Heritabilities for body length were estimated for the same breeds between 0.16 and 0.32, while in our population heritability varied between 0.52 ± 0.14 (2 months of age) and 0.26 ± 0.11 (7 months of age). Heritabilities for conformation measurements tended to be lower for measurements at 7 months of age than at 2 months of age, which may be due to a change in the ratio between phenotypic and genetic variance.

The heritability of average daily gain was 0.54 (SE = 0.14), while the heritability of pre-weaning daily gain was 0.81 (SE = 0.14) and post-weaning gain was 0.60 (SE = 0.14). Estimation of heritabilities for daily gain for different production breeds and crosses ranged between 0.20 and 0.31 (Brandt and Täubert, 1998). Kadarmideen et al. (2004) estimated heritability for post-weaning gain in the range of 0.28 for Large White and Landrace breeds, whereas Hofer et al. (1992) estimated heritabilities for total average daily gain ranging between 0.12 and 0.25. Heritabilities for DXA traits were moderate to high, with for example, a heritability of 0.57 (0.14) for the percentage of fat. Estimates of slaughter traits varied between 0.08 (SLfat_om) and 0.54 (SLcw) with high standard errors (0.07–0.15), which is due to the relatively small number of animals. Previous studies indicate high heritabilities for slaughter characteristics. For instance, Kadarmideen et al. (2004) reported a heritability of 0.60 for premium meat cuts and 0.66 for intramuscular fat, while Singh et al. (2001) reported a heritability of 0.59 for carcass weight and 0.54 for backfat thickness.

We found most estimated heritabilities to be higher than previous reports, particularly for traits studied in field populations. One of the reasons for this is that our F2 pig resource population was created with the aim to generate large genetic variability in selected traits, which will consequently increase the heritability (as well as power to detect QTLs). In addition, our results cannot be directly compared with results based on field data and production pigs.

### Genetic predictions for fatness and fasting glucose level

**Figure 4:**
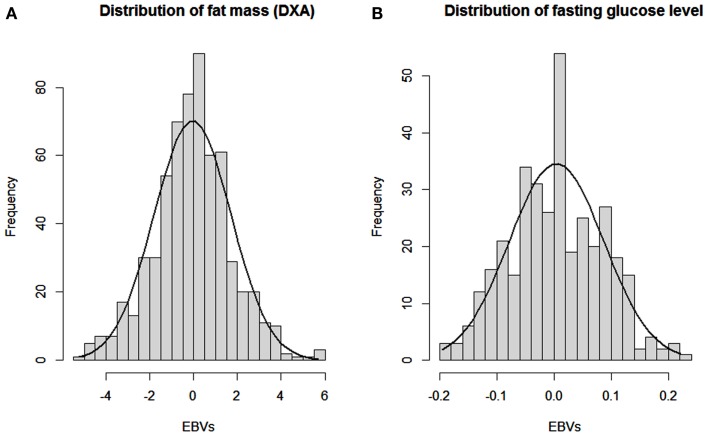
**Distributions of estimated breeding values for percentage of fat (DXA fat) and fasting glucose level (GLU)**. Distributions of estimated breeding values (EBV’s) for **(A)** percentage of fat at DXA scanning and **(B)** fasting glucose level.

The CVs show a wide range of phenotypic variation: 15–45% for traits directly related to OOR traits. Heritabilities (e.g., 0.57 for DXA%fat) showed that most of this overall variation is due to additive genetic effects, as expected from the F2 intercross. This was further investigated by plotting the distributions of estimated breeding values (EBV’s) for DXA%fat and FGL, as presented in Figure 4. It shows the wide and normally distributed genetic values within those OOR traits, resulting in some animals which are highly genetically predisposed for high or low body fatness and FGLs.

### Phenotypic and genetic correlations

A multi-trait model that analyzes several traits simultaneously (Kadarmideen et al., 2004) in a one-step estimation procedure is desirable due to the high accuracy in prediction. However this was not feasible due to singularity problems and/or illogical estimates of covariance components so we fitted a series of two-trait models. A series of two-trait or bivariate animal models yielded estimates of phenotypic and genetic correlations and reported in Tables 4 and 5. Only statistically significant genetic and phenotypic correlations are reported and discussed here onward. However, we have presented phenotypic and genetic correlations (with standard errors) between all traits (regardless of statistical significance) for the complete dataset in Table S1 in Supplementary Material.

**Table 4:** **Genetic (above the diagonal) and phenotypic (below the diagonal) correlations between DXA and weight and conformation traits with standard errors, estimated by individual animal model using all 563 animals**.

Trait^1^	DXA fat	DXA lean	DXA total	DXA%fat	WT2m	LEN2m	ABD2m
DXA fat	–	0.09 (0.07)	0.38 (0.07)	0.92 (0.01)	0.90 (0.01)	0.77 (0.03)	0.75 (0.03)
DXA lean	n.e.^2^	–	0.95 (0.01)	–0.33 (0.08)	0.97 (0.00)	0.82 (0.02)	0.89 (0.01)
DXA total	n.e.	0.97 (0.02)	–	0.31 (0.08)	n.e.	0.78 (0.03)	0.90 (0.01)
DXA%fat	0.88 (0.05)	−0.47 (0.18)	n.e.	–	0.26 (0.07)	0.18 (0.07)	0.17 (0.07)
WT2m4	0.92 (0.04)	0.97 (0.01)	n.e.	n.e.	–	0.91 (0.01)	n.e.
LEN2m	0.83 (0.08)	0.94 (0.04)	0.93 (0.04)	n.e.	0.97 (0.02)	–	0.84 (0.02)
ABD2m	0.61 (0.15)	0.97 (0.02)	0.94 (0.03)	−0.30 (0.24)	n.e.	0.87 (0.06)	–

*All presented values are significant at *p* < 0.05*.

*^1^For abbreviations and units of measurements see Table 1*.

*^2^Not estimable or estimated with no statistical significance (large s.e)*.

**Table 5:** **Genetic correlations of slaughter traits with weight and conformation with standard errors, estimated by individual animal model using all 563 animals**.

Trait^1^	WT2m	WT7m	LEN2m	LEN7m	ABD2m	ABD7m	ADG	BMC
SLcw	0.32 (0.23)	0.90 (0.07)	n.e.^2^	n.e.	n.e.	0.37 (0.27)	0.96 (0.03)	0.45 (0.21)
SLmeat	0.74 (0.28)	n.e.	0.71 (0.28)	n.e.	n.e.	n.e.	0.66 (0.22)	n.e.
SL%meat	−0.72 (0.23)	−0.58 (0.32)	−0.72 (0.27)	−0.56 (0.31)	−0.43 (0.35)	−0.38 (0.33)	n.e.	−0.86 (0.14)
SLfat	n.e.	0.60 (0.22)	n.e.	n.e.	n.e.	0.66 (0.23)	0.74 (0.14)	n.e.
SLbf1	0.57 (0.24)	0.65 (0.27)	0.56 (0.25)	n.e.	0.50 (0.26)	0.42 (0.36)	0.47 (0.29)	0.75 (0.20)
SLom_fat	n.e.	0.53 (0.23)	n.e.	n.e.	−0.32 (0.29)	0.36 (0.30)	0.71 (0.16)	n.e.

*All presented values are significant at *p* < 0.05*.

*^1^For abbreviations and units of measurements see Table 1*.

*^2^Not estimable or estimated with no statistical significance (large s.e)*.

Weight measurements show a high genetic correlation with conformation measurements (0.36–0.99) and DXA scanning results, as expected (Table 4). Previous research also indicated high correlations (0.51–0.75) between length and weight, for different production breeds (Johnson and Nugent, 2003).

In human research, BMI is popular due to simplicity of measurement and proven association with obesity. However, it is also known that BMI has many shortcomings (Heber, 2010). For example, ethnic differences and the influence of muscle mass (athletes) on weight result in misdiagnosis and misunderstanding of obesity. BAI is proposed as a better index for body adiposity (Bergman et al., 2011). BAI is expected to be a better indicator of per cent adiposity than measurements of body weight. However, critics raise the question whether this measure solves above-mentioned problems with BMI (Heymsfield and Shen, 2011). Heritability estimates for BMI and BAI are comparable, 0.56 and 0.58 respectively. To evaluate both measurements in order to determine which measurement would explain most about the measurements at slaughter, genetic correlations between BMI/BAI and slaughter traits were assessed. As expected, BAI in general gives a more negative genetic correlation with slaughter traits than BMI, e.g., 0.72 vs. −0.49 for meat percentage and −0.79 vs. 0.54 for backfat (mm). This stronger relationship indicates that BAI is a better indicator for adiposity than BMI. These results are novel, as to date no other studies have so far evaluated correlations between these traits.

Previous research has indicated a negative correlation between extreme obesity and BMD as well as between extreme obesity and BMC (Weiler et al., 2000). This correlation is mainly seen in postmenopausal women, resulting from decreased production of ovarian estrogens (Nunez et al., 2007), and is less strong in men than in women (Felson et al., 1993), resulting in a higher BMD for men (Arabi et al., 2004; Brown et al., 2004). In this F2 resource population, no significant difference between males and females was found (*P* = 0.09); however, females have a slightly higher BMC and BMD than males, which is not in line with human research. Conversely, a study in a Duroc × Pietrain crossbred population shows no gender effect for BMD and BMC (Laenoi et al., 2011). Strong positive genetic correlations were found between BMC and fat mass indicated by DXA scanning (0.85, SE = 0.06) and the same was evident for genetic correlations with backfat (mm) at slaughtering (0.89, SE = 0.15). Since obesity causes a lower BMD and BMC in humans, these results are not in line with the expected negative correlation. A possible explanation for these findings is the fact that measurements in our study are made on pigs at a very young age and thus, the results are not comparable to human studies.

It is recognized that adipose tissue is a highly active metabolic and endocrine organ, and dysfunction of adipose tissue is associated with insulin resistance (Goossens, 2008). This resource population has a wide variation in adiposity, and consequently it can be expected that there is also a wide variation in the level of metabolic parameters affected by obesity. As mentioned previously, pigs are a good model for human type 2 diabetes (Bellinger et al., 2006). In humans normal fasting plasma glucose levels are below 5.6 mmol/L, impaired plasma glucose levels are between 5.6 and 7 mmol/L, while type 2 diabetes has plasma glucose levels above 7 mmol/L. Previous research indicated lower fasting plasma glucose levels for the minipig (i.e., 3.6 mmol/L Larsen et al., 2003), whereas the domestic pig is estimated at the same level as humans (Larsen and Rolin, 2004). In our resource population, FGLs range between 1.80 and 11.15, with an average of 4.50 (SE = 2.18). The high heritability of FGL in this population (0.49) shows that there is a clear genetic background for FGLs in this population. There is a scarcity of research published on the genetics of FGLs in pigs, but our estimate indicates that glucose metabolism is heritable in pig populations. Studies of heritability for FGLs in humans show heritabilities between 0.38 and 0.51 for twin studies in Northern Europe, 0.08–0.72 for family studies and around 0.25 after adjustments in a genetically isolated population (Santos et al., 2006). Genetic parameter estimations show a positive correlation with weight (0.61), length (0.57), abdominal circumference (0.41), and fat mass at DXA scanning (0.59). The genetic correlations with the fat characteristics at slaughtering show a strong positive correlation (0.90), but a strong negative correlation with the meat characteristics (−0.56 to −0.76). As expected, these results show an elevated plasma glucose level with higher fat mass in the F2 population, which may indicate impaired glucose metabolism in some of the animals in this population (Goossens, 2008).

At approximately 100 kg, the pigs were slaughtered and dismembered. Weight characteristics show a positive genetic correlation with fat characteristics (between 0.40 and 0.76), and a negative genetic correlation with meat characteristics (between −0.45 and −0.76), which shows that an increase in weight of the pigs mainly results in an increase of fat (Table 5). The same was the case for conformation measurements: an increase in length mainly results in increase of backfat (0.56, SE = 0.25), and an increase of abdominal circumference results in increase of fat mass (0.66, SE = 0.23). Previous research indicated a positive correlation between length and backfat, between 0.10 and 0.41 for different production breeds (Johnson and Nugent, 2003), which is lower than estimates in this study (0.56, SE = 0.25). As mentioned earlier, the advantage of this F2 resource population is the possibility of measuring traits which are complicated or expensive to measure in human studies. One of these traits is the average daily gain. In this study strong genetic correlations are found between ADG and fat characteristics at slaughtering, e.g., between ADG and SLfat 0.74 (0.14). These results show that a strong growth in this F2 resource population mainly results in the accumulation of fat mass, as the genetic correlation with lean meat is negative (e.g., between ADGII and SL%meat −0.33, SE = 0.29). As expected on the basis of earlier mentioned correlations, a strong positive genetic correlation is found between fat characteristics at slaughtering and BMC (0.75, SE = 0.20) and the FGL (0.39, SE = 0.32).

In the current paper we describe a unique resource population consisting of 450 F2 pigs created by using divergent breeds (Yorkshire/Duroc × Göttingen Minipig) as founders. The population was designed with the purpose of establishing a pig model in which the genetic background of OOR and associated traits can be studied. The statistical analyses made on the phenotypic traits recorded in the population clearly demonstrate that potential QTL’s causing variation in the traits of interest are likely segregating in the F2 pig resource population. This study considered a total of 563 animals (454 with phenotypes and others are F0 and F1 parental animals) for estimation of genetic parameters. While this may be argued as low number of animals for parameter estimation, several other studies used fewer number of animals (fewer than 563 animals used in this study) to estimate genetic parameters, e.g., by Van Der Waaij et al. (2003). The established F2 population was measured for phenotypes with expected heritabilities in the range of around 0.25 to 0.50. From power calculations based on this assumption of heritabilities, we have concluded that around 450 F2 animals would be sufficient to detect even small QTLs explaining only around 2% of genetic variance at 80% power. Thus, genes involved in the phenotypes measured within the population can be identified in future genomic and systems genetic studies. This is also supported by previous QTL mapping studies using far fewer number of F2 animals (e.g., Ovilo et al., 2002; Hanotte et al., 2003). The whole resource population has been genotyped using the 60 K SNP Chip, which will be used in genome-wide association and fine QTL mapping studies as parts of the BioChild (www.biochild.ku.dk) and UNIK projects. Moreover, several tissues were collected at slaughtering, of which some will be used for RNA-sequencing to study transcriptomic profiles in extremely obese vs. lean pigs, as identified by the quantitative genetic analysis results reported here. In conclusion, the creation of this pig resource population resulted in a great potential to study the genetic architecture of OOR diseases.

## Conclusion

Quantitative trait loci mapping or GWAS requires a thorough knowledge on genetic variances and any (genetic) relationships amongst traits that are investigated, particularly if such parameters for novel traits have not yet been reported elsewhere. This study has contributed to creating such knowledge for the established F2 pig resource population and provided a strong support for genomic investigations of OOR traits via GWAS and/or QTL mapping. Specifically, high heritabilities and strong correlations between weight, conformation, DXA, and slaughter traits between the different traits were reported here. This study, to the best of our knowledge, is the first experimental study in pigs in which a wide range of obesity traits, including novel DXA scan traits, information on different fatness traits at slaughter, and FGL have been measured in a large number of experimental animals. Consequently, it is the first study to report quantitative genetics characteristics on these novel OOR traits (in terms of estimated heritabilities and genetic correlations). In conclusion, the established F2 population gives a good rationale for further genome-wide and systems genetics approaches for detecting DNA variants, pathways, and gene networks affecting human obesity.

## Authors’ Contribution

Merete Fredholm was the experimental project leader who originally conceived the F2 experiment and collected/supervised the measurements on all pigs. Haja N. Kadarmideen was the statistical genetics analysis project leader who originally conceived the quantitative genetics design, models, and analysis part and supervised Lisette J. A. Kogelman in the analyses. Lisette J. A. Kogelman collated, edited, and analyzed the data. Lisette J. A. Kogelman wrote the first draft of the manuscript with contributions from Haja N. Kadarmideen, Thomas Mark, and Merete Fredholm. Peter Karlskov-Mortensen, Camilla S. Bruun, Susanna Cirera, Thomas Mark, Mette J. Jacobsen, and Claus B. Jørgensen contributed to pedigree development and phenotypic measurements of the pigs. All authors read and approved the final version of the manuscript.

## Conflict of Interest Statement

The authors declare that the research was conducted in the absence of any commercial or financial relationships that could be construed as a potential conflict of interest.

## Supplementary Material

The Supplementary Material for this article can be found online at http://www.frontiersin.org/Livestock_Genomics/10.3389/fgene.2013.00029/abstract

Supplementary Table S1 Phenotypic and genetic correlations of all analyzed variables. Excel file including all phenotypic and genetic correlations, with their standard errors.

## Appendix

**Table A1:** **Solutions, standard errors, and *p*-values for all fixed effects in the univariate analysis**.

	Variable	Trait*	Age**		Sex***			SOB****				Length	
		μ (SE)	μ (SE)	*p*-value	Male (SE)	Female (SE)	*p*-value	1 (SE)	2 (SE)	3 (SE)	*p*-value	μ (SE)	*p*-value
**WEIGHT TRAITS**													
	At birth	0.83 (0.04)	–	–	0.00 (0.00)	−0.02 (0.01)	0.086	0.00 (0.00)	−0.00 (0.07)	0.01 (0.08)	0.979	–	–
	At 2 weeks	2.73 (0.12)	–	–	0.00 (0.00)	−0.04 (0.04)	0.335	0.00 (0.00)	−0.03 (0.20)	0.06 (0.23)	0.945	–	–
	At 5 weeks	5.50 (0.47)	–	–	0.00 (0.00)	0.22 (0.11)	0.039	0.00 (0.00)	0.77 (0.71)	0.80 (0.76)	0.475	–	–
	At ± 2 months	−6.77 (1.98)	0.29 (0.03)	<0.001	0.00 (0.00)	0.63 (0.29)	0.018	0.00 (0.00)	1.36 (1.61)	0.04 (1.73)	0.319	–	–
	At ± 125 days	3.726 (5.95)	0.28 (0.05)	<0.001	0.00 (0.00)	2.84 (0.80)	0.005	0.00 (0.00)	3.23 (4.69)	−3.02 (6.24)	<0.001	–	–
	At ± 7 months	51.33 (5.09)	0.18 (0.02)	<0.001	0.00 (0.00)	1.36 (1.52)	0.074	0.00 (0.00)	4.25 (4.73)	7.78 (5.17)	0.068	–	–
Length	At ± 2 months	28.23 (2.57)	0.31 (0.03)	<0.001	0.00 (0.00)	1.22 (0.45)	0.004	0.00 (0.00)	1.67 (1.96)	−0.76 (2.12)	0.316	–	–
	At ± 7 months	78.11 (1.95)	0.03 (0.00)	<0.001	0.00 (0.00)	−1.82 (0.61)	<0.001	0.00 (0.00)	1.45 (1.68)	1.49 (1.85)	0.387	–	–
Abd. Circ	At ± 2 months	29.45 (3.06)	0.47 (0.04)	<0.001	0.00 (0.00)	1.04 (0.54)	0.033	0.00 (0.00)	0.62 (2.31)	−1.26 (2.50)	0.576	–	–
	At ± 7 months	100.70 (3.06)	0.08 (0.01)	<0.001	0.00 (0.00)	5.43 (0.97)	<0.001	0.00 (0.00)	0.16 (2.58)	5.34 (2.84)	0.048	–	–
Shoulder height	At ± 2 months	23.71 (1.97)	0.25 (0.03)	<0.001	0.00 (0.00)	0.19 (0.30)	0.419	0.00 (0.00)	0.61 (1.57)	−0.98 (1.68)	0.438	–	–
	At ± 7 months	58.87 (2.37)	0.04 (0.01)	<0.001	0.00 (0.00)	−1.52 (0.60)	<0.001	0.00 (0.00)	−0.40 (1.55)	0.00 (0.00)	0.464	–	–
Thorax height	At ± 2 months	14.60 (1.65)	0.13 (0.02)	0.677	0.00 (0.00)	0.38 (0.28)	<0.001	0.00 (0.00)	1.02 (1.27)	−3.97 (1.36)	0.002	–	–
	At ± 7 months	38.22 (1.55)	0.06 (0.00)	<0.001	0.00 (0.00)	−0.69 (0.46)	<0.001	0.00 (0.00)	2.65 (1.46)	2.78 (1.59)	0.020	–	–
**DAILY GAIN**													
	Average	0.42 (0.02)	–	–	0.00 (0.00)	0.03 (0.00)	<0.001	0.00 (0.00)	−0.00 (0.03)	0.00 (0.03)	0.978	–	–
	Pre-weaning	0.18 (0.02)	–	–	0.00 (0.00)	0.01 (0.00)	0.009	0.00 (0.00)	0.02 (0.03)	0.00 (0.03)	0.818	–	–
	Post-weaning	0.53 (0.04)	–	–	0.00 (0.00)	0.05 (0.01)	<0.001	0.00 (0.00)	−0.02 (0.05)	−0.02 (0.06)	0.924	–	–
BMI	At ± 2 months	24.67 (3.26)	0.42 (0.04)	<0.001	0.00 (0.00)	−0.16 (0.55)	0.902	0.00 (0.00)	−0.59 (2.51)	1.11 (2.70)	0.807	–	–
	At ± 7 months	92.98 (6.08)	0.16 (0.02)	<0.001	0.00 (0.00)	7.84 (1.95)	0.021	0.00 (0.00)	1.79 (5.00)	6.07 (5.52)	0.210	–	–
BAI	At ± 2 months	0.20 (0.00)	0.00 (0.00)	0.053	0.00 (0.00)	0.01 (0.00)	0.010	0.00 (0.00)	−0.00 (0.00)	0.00 (0.00)	0.334	–	–
	At ± 7 months	0.15 (0.00)	0.00 (0.00)	0.162	0.00 (0.00)	0.01 (0.00)	<0.001	0.00 (0.00)	−0.00 (0.00)	0.00 (0.00)	0.540	–	–
DXA traits	Fat mass	−14.13 (2.52)	−0.03 (0.03)	<0.001	0.00 (0.00)	−0.04 (0.04)	<0.001	0.00 (0.00)	−0.84 (1.58)	0.40 (1.72)	0.292	1.31 (0.04)	<0.001
	Lean mass	−42.13 (3.11)	0.39 (0.04)	<0.001	0.00 (0.00)	−0.57 (0.49)	<0.001	0.00 (0.00)	−0.65 (2.06)	1.38 (2.23)	0.016	2.39 (0.05)	<0.001
	Total mass	−44.16 (3.31)	0.34 (0.04)	<0.001	0.00 (0.00)	−0.55 (0.56)	<0.001	0.00 (0.00)	−0.89 (2.07)	1.36 (2.24)	0.016	2.73 (0.06)	<0.001
	Fat (%)	5.06 (0.16)	−0.01 (0.00)	<0.001	0.00 (0.00)	0.02 (0.03)	0.609	0.00 (0.00)	0.01 (0.13)	−0.04 (0.14)	0.911	–	–
													
	BMC	4.61 (0.15)	0.02 (0.00)	<0.001	0.00 (0.00)	0.04 (0.02)	0.070	0.00 (0.00)	0.05 (0.12)	0.07 (0.13)	0.702	–	–
	BMD	−1.12 (0.07)	0.00 (0.00)	<0.001	0.00 (0.00)	0.02 (0.01)	0.067	0.00 (0.00)	0.02 (0.06)	0.05 (0.07)	0.652	–	–
													
Glucose	fasting level	1.91 (0.16)	−0.00 (0.00)	0.715	0.00 (0.00)	0.07 (0.05)	0.015	0.00 (0.00)	1.01 (0.11)	0.00 (0.00)	<0.001	–	–
**SLAUGHTER TRAITS**													
	Weight leaf fat	−3.48 (0.69)	0.00 (0.00)	<0.001	0.00 (0.00)	0.43 (0.09)	0.042	0.00 (0.00)	0.46 (0.23)	0.85 (0.26)	0.002	0.05 (0.00)	<0.001
	Meat (%)	47.64 (2.08)	−0.00 (0.00)	0.677	0.00 (0.00)	−3.51 (0.67)	<0.001	0.00 (0.00)	−3.96 (1.23)	−6.81 (1.45)	0.002	–	–
	Backfat position 1	34.26 (2.94)	−0.02 (0.01)	0.055	0.00 (0.00)	3.15 (0.92)	<0.001	0.00 (0.00)	2.65 (1.87)	4.87 (2.17)	0.189	–	–
	Backfat position 2	26.22 (2.77)	0.02 (0.01)	0.092	0.00 (0.00)	4.54 (0.90)	<0.001	0.00 (0.00)	6.03 (1.52)	9.89 (1.84)	<0.001	–	–
	Meat (mm)	50.55 (2.76)	0.01 (0.01)	0.274	0.00 (0.00)	−2.48 (0.86)	0.001	0.00 (0.00)	0.52 (1.80)	−1.61 (2.09)	0.636	–	–
	Carcass weight	31.75 (3.53)	0.08 (0.01)	<0.001	0.00 (0.00)	1.73 (1.01)	0.683	0.00 (0.00)	9.59 (3.00)	12.97 (3.28)	<0.001	–	–
	Omental fat	−283.79 (107.05)	0.98 (0.19)	<0.001	0.00 (0.00)	90.56 (15.56)	<0.001	0.00 (0.00)	91.88 (37.03)	188.64 (43.28)	0.004	3.40 (1.15)	0.004
	Intestinal fat	3.60 (7.74)	0.04 (0.01)	0.005	0.00 (0.00)	2.80 (1.19)	0.018	0.00 (0.00)	3.37 (1.38)	4.70 (1.95)	0.205	0.03 (0.09)	0.744

**All estimates of μ are significant (*p*-value = <0.001)*.

***Age at relevant time moment*.

****The gender effect is set to zero for the males, whereby the females are a deviation of the mean*.

*****The season of birth (SOB) effect is set to zero for SOB 1, whereby SOB 2 and 3 are deviations from the mean*.
